# Treatment and Outcomes of Tusk Fractures in Managed African Savanna and Asian Elephants (*Loxodonta africana* and *Elephas maximus*) across Five Continents

**DOI:** 10.3390/ani12091125

**Published:** 2022-04-27

**Authors:** Josephine B. Rose, Austin Leeds, Linda M. Yang, Rachel LeMont, Melissa A. Fayette, Jeffry S. Proudfoot, Michelle R. Bowman, Allison Woody, James Oosterhuis, David A. Fagan

**Affiliations:** 1 Avian and Exotic Veterinary Care, Portland, OR 97213, USA; 2 Oregon Zoo, Portland, OR 97221, USA; 3 Disney’s Animal Kingdom ®, Lake Buena Vista, FL 32830, USA; austin.leeds@disney.com; 4 VCA Rock Creek Animal Hospital, Aloha, OR 97006, USA; lindameowsyang@gmail.com; 5 College of Veterinary Medicine, Kansas State University, Manhattan, KS 66502, USA; rlemont@vet.k-state.edu; 6 Indianapolis Zoological Society, Indianapolis, IN 22309, USA; mfayette@indyzoo.com (M.A.F.); jproudfoot@indyzoo.com (J.S.P.); mbowman@indyzoo.com (M.R.B.); 7 The Colyer Institute, San Diego, CA 92196, USA; woodydvm@yahoo.com (A.W.); jeo.dvm@cox.net (J.O.); colyer@pacbell.net (D.A.F.)

**Keywords:** *Loxodonta* sp., *Elephas* sp., tusk fracture, endodontics, partial pulpotomy, pulpitis, tusk extraction

## Abstract

**Simple Summary:**

When tusk fractures occur in elephants under managed care, they can expose the soft tissues and substantial blood supply of the tusk’s pulp. The management strategies and clinical consequences of those fractures vary immensely in both the literature and the collective experiences of elephant managers and clinicians. Outcomes have ranged from self-healing with conservative management to life-threatening systemic infection. A detailed survey requesting tusk fracture characteristics and subsequent management and outcomes was completed by elephant veterinarians globally. A larger pulp canal diameter at the time of fracture and the use of tap water in the course of treatment were associated with an increased risk of the development of an infected and inflamed pulp, meriting further investigation. Endodontic treatment of fractured tusks with pulp exposure was associated with a reduced risk of tusk extraction. These guidelines can aid elephant managers and clinicians in their clinical decision making surrounding these challenging events.

**Abstract:**

Elephant tusk fractures are a clinical challenge that can impact the overall health of the animal, particularly when they result in pulp exposure. An international survey was sent to veterinarians to understand individual fracture characteristics and management strategies as they relate to outcomes, with the goal of better informing treatment procedures. The data set consisted of 79 fractures from 64 elephants (including Asian and African males and females), 44.3% of which were Class III fractures with pulpal involvement. Of this subset, pulp canal exposures of >0.5 cm were 23.8-fold more likely to develop pulpitis than fractures with <0.5 cm exposed, though canal size did not impact healing versus extraction outcome. Odds ratios showed that treatments including endodontics were 12.0-fold more likely to heal than tusks treated exclusively with medical management, though no association was observed in reducing the risk of pulpitis. Further, pulpitis was 7.58-fold more likely to develop when tap water was used to rinse exposed pulpal tissue; a finding that merits further investigation. The use of endodontic treatment versus medical management alone was significantly associated with improved recovery outcomes (i.e., reduced risk of extraction) in tusk fractures with pulpal involvement.

## 1. Introduction

Traumatic tusk fractures that occur in managed elephants present a serious clinical challenge to veterinarians and elephant care staff. Fractures with pulp exposure can result in ascending bacterial infection and/or inflammation of the dental pulp [1,2,3]. Uncontrolled pulpitis can result in a non-viable tusk that ultimately requires extraction [1] and has been reported as a cause of sepsis and death [4]. Pain, particularly if there is extension of pathology to the periodontal ligament and/or the pulpal apex, can occur [5,6,7]. The management of fractures can be technically and logistically challenging to coordinate and can require anesthetic events [8]. Elephant anesthesia requires specialized knowledge, with 5 of 159 reported elephant deaths from 1988 to 1999 being associated with anesthetic or perianesthetic events [9]. Further, even if tusk extraction is pursued, it may take several procedures to successfully resolve infection and fully remove the tusk if severe apical disease is present, particularly if tertiary dentin ankyloses into the surrounding alveolar bone [10,11,12].

While multiple reports describe various tusk repair and extraction techniques [1,3,6,13,14], the decision of whether to preserve or extract a damaged tusk is based on prior clinical experience of dental specialists [1,11]. A decision matrix has been developed to guide the clinician in cases of tusk pulp exposure, emphasizing the importance of removing diseased pulpal tissue and performing endodontic care, to optimize outcomes [11]. This matrix has been utilized successfully within institutions [6]. Clinical decision making in regard to tusk fractures, particularly those that involve exposure of the pulp, warrants further investigation. The extensive blood supply of elephant tusks can facilitate remarkable healing or alternatively serve as a nidus for severe ascending infection [4,15]. An endodontic approach that preserves the viability of the tusk pulp while protecting the pulp from further contamination represents “best practice’’ in tusk fracture management [11,16]. A critical evaluation of tusk fracture circumstances, management techniques and treatment outcomes should be a highly valuable addition to the above-mentioned case and technique papers, contributing to guidelines for clinical case best practices. Particularly, a multi-institutional approach to such an evaluation should be valuable considering the logistical challenges and specialized veterinary skills required to treat fractures and because reports have suggested tusk fractures can heal with comparatively conservative approaches and/or limited human intervention [17,18,19].

The aim of this research is to better understand how elephant tusk fractures can be optimally managed by establishing relationships between variables and tusk fracture outcomes. Deeper understanding of these relationships will aid veterinarians and dental specialists in their management strategies for these challenging clinical cases.

## 2. Materials and Methods

This study’s research aim was met by developing a survey (available for download in Supplementary Materials) that aided in achieving five research objectives. First, to determine the individual characteristics of elephants that fractured their tusks including the species of elephant, gender, age of elephant at the time of fracture, and elephant weight in kilograms at the time of fracture. Elephants within this study were classified according to age class, based on reported descriptions of age in relation to tusk development [20]. This metric was developed for wild African elephants, and a similar metric has not been reported for Asian elephants to the authors’ knowledge. This classification of the data was performed to illustrate potential variation of tusk development within the study population and to investigate any links to fracture outcomes. The authors acknowledge that Asian elephant females’ tusks are either absent or colloquially referred to as “vestigial,” rarely extending beyond the gingival sulcus, but they are fully developed tusks when present [2,11]. For this reason, they have been included within the analysis, though supplemental separate statistics were run if there was an indication that the size dimorphism could be impactful to the interpretation of results. 

The second objective was to describe the characteristics of tusk fractures that have occurred in elephants under managed care. This section of the survey asked for the side of tusk affected, the approximate length of tusk that was fractured, whether a previous tusk fracture had occurred on the tusk, whether pericoronitis was present, whether pulpal tissue was suspended from the fracture site, the diameter of pulp canal exposed, the proximal extent of the fracture, the presence of reparative dentin within the tusk pulp at the time of fracture, if any disruption of the periodontal ligament was evident, and whether clinical evidence of infection existed at the time of fracture. Further definitions are available in Table 1.

**Figure 1:**
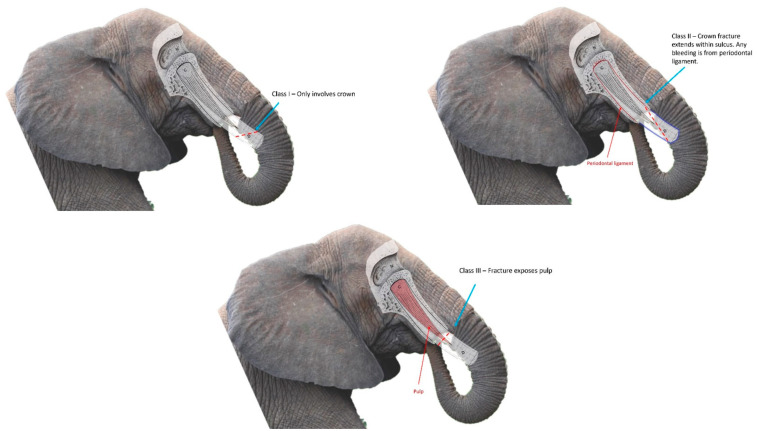
Diagrams illustrating tusk fracture classification system utilized in survey. Diagram modified from previous publication with permission [15].

For the purposes of this study, fractures were categorized as Class I fractures (involvement of the crown exclusively), Class II fractures (crown fracture leading to disruption of the periodontal attachment), and Class III fractures (involving the dental pulp). The American Veterinary Dental College (ADVC) has defined similar types of fractures in brachydont teeth as Uncomplicated Crown Fractures (UCF), Uncomplicated Crown Root Fractures (UCRF), and Complicated Crown Fractures (CCF), respectively [21]. Respondents were further asked to classify any fracture that ADVC would otherwise define as a Complicated Crown Root Fracture (CCRF) as a Class III fracture, similar to a CCF. The reasoning for combining CCFs and CCRFs into Class III fractures was twofold—first, to streamline data analysis. Second, to account for the degree of uncertainty for root involvement in an elephant tusk fracture, due to the challenges of penetrating the alveolar bone with radiography, and thus confirming root involvement, in elephants [11]. While the Class I–III system employed within the survey incorporates definitions with the AVDC tooth fracture classification, elephant tusks are elodont incisors that differ in structure [22] from brachydont teeth. Diagrams with corresponding descriptions were embedded within the survey to clarify this classification system (Figure 1). 

The third objective was to report any medical management strategies employed for the tusk fracture. A section was developed asking which topical and systemic medical treatments were utilized for the fracture. For this section, the following was asked for topical treatments applied to the pulp, antibiotics administered, and non-steroidal anti-inflammatories (NSAIDs) administered: select all treatments utilized from a list, record the period of time from onset of fracture to onset of a treatment, duration of treatment, and dosing. For antibiotics, it was additionally asked if any resistance was confirmed for the antibiotic choice and the reason for discontinuation of this treatment. The exact treatments requested in the survey are available in Supplementary Materials. 

The fourth objective sought to describe endodontic management strategies. Questions were included about attempts to protect the exposure of the pulp, the materials used, and the time from fracture development to the accomplishment of these attempts. It further requested information about to what degree diseased pulp could be removed prior to covering the exposed pulp, how hemostasis was achieved, what disinfecting agents were utilized, and what the composition of any pulp dressing, restoration, and/or materials used in a crown. The exact materials referenced are available in Supplementary Materials. The information gathered through meeting these four objectives became the “variables” within this study. Though details of both medical and endodontic management strategies were asked to be recorded, broader categorizations of strategies for management were developed as “medical management only” and “medical management combined with endodontic management”.

Medical management included all interventions described in association with Objective 3. Endodontic management included all strategies with the goal of protecting the exposed pulp, either via a temporary cap, composite and/or partial pulpotomy, as obtained through Objective 4. The fifth and final objective was to classify subsets of tusk outcomes and relate those outcomes to the variables. Respondents were asked to choose all relevant listed outcomes of that tusk’s fracture from a list and were also allowed to elaborate further if necessary. These outcomes were specific (Table 2), primarily pertaining to whether pulpitis had ever developed and/or if the tusk developed complications necessitating extraction. In the analysis, “pulpitis” versus “no pulpitis” and “tusk fully healed and remains viable” versus “tusk developed complications necessitating extraction” were analyzed separately so that each variable had the potential to be evaluated under both metrics. Clinical definitions of pulpitis and resolution of pulpitis were frequently based on clinical impression (Table 2). 

Prior to distributing the survey to institutions, this study was endorsed by the Association of Zoos and Aquariums (AZA) Elephant Taxon Advisory Group and Species Survival Plan in addition to the European Association of Zoos and Aquaria (EAZA) Elephant Taxon Advisory and Research Groups. The survey was translated from English to Japanese by Yurie Yamada, BVSc, facilitating the participation of elephant managers in the Japanese Association of Zoos and Aquariums (JAZA). Invitations were sent to elephant managers in North America, South America, Europe, Asia and Australia via listservs and direct email communication with known elephant managers. Each participating institution reviewed and approved the survey methodology prior to completion. Both EAZA and AZA research proposals and endorsements, including all survey questions, were provided for review via email at the time of invitation. If requested, further research approval agreements were achieved at the individual institution level. Several, but not all, institutions provided medical records for further review. The survey was designed so that only the most recent fracture of each tusk would be a data point, allowing one elephant to be represented up to two times in the data set. Information from the surveys was augmented with information provided through the AZA, EAZA and JAZA studbooks for both African and Asian elephants as needed. This study was primarily limited to fractures that occurred from 2009 onward, but fractures prior to this requested time frame were included if they had sufficient information to complete the survey. The survey was developed through a survey platform that is authorized for use in research [23].

Data from the survey were summarized with descriptive statistics focused on intraspecific sex comparisons, frequency of tusk fracture covariates, and treatment outcomes. Inferential statistical analysis of these count data was completed via Chi Square tests. Not all descriptive comparisons included a complementary inferential analysis as the distribution of our count data did not always meet the assumptions of the Chi Square test. Inferential analysis was conducted via SPSS V.24 (IBM, Armonk, NY, USA) with significance set to 0.05. Descriptive statistics were conducted in Excel (Microsoft, Redmond, WA, USA).

## 3. Results

Data were contributed by 35 institutions across 10 countries including Australia, Belgium, Colombia, Germany, Ireland, Japan, Mexico, Netherlands, Switzerland, the United Kingdom, and the United States.

### 3.1. Individual Elephant Characteristics

This data set consisted of 79 fractures from 64 individuals (n_African_ = 18.9; n_Asian_ = 23.14). Details of the fractures included in the data set are provided in context of the age of the elephants at the time of fracture, the species of elephant, and weights of the elephants at the time of fracture (Table 3). 

### 3.2. Characteristics of Fractured Tusks 

The key highlights of the analyses are summarized (Table 4). Of the 79 tusk fractures, 41 involved the right tusk (51.9%; n_African_ = 10.7, n_Asian_ = 13.11) and 36 involved the left tusk (45.6%; n_African_ = 11.4; n_Asian_ = 13.8). Two fractures (2.5%) did not have a side specified. Length of tusk at time of fracture varied, with 45.6% of fractures occurring in tusks less than 30 cm (n_Total_ = 36, n_African_ = 8.5; n_Asian_ = 9.14), 12.7% in tusks 30 to 60 cm (n_Total_ = 10, n_African_ = 3.2; n_Asian_ 5.0), and 17.7%% in tusks greater than 60 cm (n_Total_ = 14, n_African_ = 6.0; n_Asian_ = 8.0) in length. Nineteen (24.0%) fractures did not have a tusk length reported at the time of fracture.

Of all reported fractures, 36.7% were considered Class I, 12.6% were Class II, and 44.3% were Class III. Two “fractures” (2.5%) were not fractures but complete avulsions from the alveolar bone, and both occurred in Asian elephant females. One fracture (1.3%) did not have a class reported, and three fractures (3.8%) from Asian elephant females could not be classified as Class II or III based the challenges in visualizing the tusk deep within the gingival sulcus. More than one-third (38.0%) of all reported fractures occurred in tusks that had been fractured previously. When combining males and females of both species, no association was observed between species and proximity of fracture to gingival crest (χ2 = 0.361, df = 1, P = 0.548). When Asian elephant females were removed from this analysis due to their sexually dimorphic tusks, there was still no significant association (Table 4). There was a descriptive difference, however, with 64.0% of African elephant and 38.1% of Asian male elephant tusk fractures occurring within 5 cm of the gingival crest at the proximal aspect; 100% of Asian female fractures occurred within 5 cm of the gingival crest. The presence of pericoronitis was observed in only 11.4% of all fractures, and was noted in both of the Asian elephant female tusks that fully avulsed. Disruption of the periodontal ligament was reported in one fracture among the African elephants. 

This occurred in a female juvenile with steadily growing tusks. Periodontal ligament disruption was not reported in any additional Asian elephants beyond the two females with the tusk avulsions (4.3% of Asian elephant tusk fractures), and both of these females were over 30 years of age. These three cases that exhibited periodontal ligament disruption accounted for only 3.8% of the total number of fractures. Reparative dentin occurred in almost one-fifth (Table 4) of African elephant tusk fractures, but was not reported in any Asian elephant tusk fractures.

Neither pulpitis nor extraction were confirmed in association with Class I or Class II fractures. Thus, the subsequent summary and analysis focus on the variables and outcomes of Class III fractures. As no confirmed Class III fractures occurred in Asian elephant females, they were not included in any outcome-based analyses. 

### 3.3. Elephant and Fracture Characteristics and Outcomes

A significant association was found between the species of elephant and the occurrence of Class III tusk fractures. The odds ratio showed Class III fractures were 7.0-fold more likely to occur in African elephants than Asian elephants. This finding remained consistent even when Asian elephant females were removed from the analysis (χ2 = 5.577, df = 1, P = 0.032), and all African elephants were 3.63-fold more likely to incur a Class III fracture compared to Asian elephant males. Overall, Class III fracture shape was similarly distributed between oblique (44.4%) and transverse forms (55.6%). Fracture shape and species had no association (χ2 = 1.543, df = 1, P = 0.357).

No relationship was found between the developmental stage of the elephant (both species combined) and the development of pulpitis in a fracture. Pulpitis developed in 42% of non-adult/juvenile fractures and 67% of subadult and adult fractures. Further, no significant relationship was noted between the age of the elephant and whether the tusk healed with medical management only, as this occurred in 17.6% of newborn to juvenile-aged elephants, and 44.4% of subadult to adult-aged elephants.

The size of pulp canal exposure varied more for African elephants than Asian elephants. For African elephants, 57.9% of canal exposures were ≤1 cm, 10.5% were 1–2 cm, and 31.6% were >2 cm, whereas 100% of Asian elephants had an exposure of ≤1 cm. The size of pulp canal exposed and the development of pulpitis had a significant relationship, with odds ratios showing that fractures with pulp canal exposures of greater than 0.5 cm were 23.8-fold more likely to develop pulpitis than fractures with less than 0.5 cm exposed. No association existed between the size of the pulp canal exposed and tusk viability outcome. Pulpal tissue was suspended from the fracture site in one-third of cases and with no association with species (χ2 = 0.300, df = 1, P = 0.690). The presence of suspended pulp tissue from fracture site was not associated with what treatment type (endodontic versus medical management only) was pursued (χ2 = 0.554, df = 1, P = 0.457), viability outcome, nor the occurrence of pulpitis. 

Evidence of pulp infection occurred in 31 of the 60 cases, with varied clinical signs including elevated white blood cell count, foreign debris present in the fracture site, grossly evident purulent discharge/necrosis of pulp, positive biopsy confirming infection, gas tracts in pulp, and soft tissue swelling near the orbit. The most commonly reported of these was grossly evident purulent discharge/necrosis of pulp, observed in 38.7% of the reports. Microorganism presence was also highly variable by case (Table 5).

A relationship between evidence of infection within the pulp at the time of fracture (parameters outlined in Table 1) and development of pulpitis was established (results summarized in Table 4). Fractures with local infection developed pulpitis in 88% of cases, whereas only 10% of those with no evidence of local infection developed pulpitis. Fractures with evidence of local infection were also 12-fold more likely to result in extraction than fractures with no evidence of local infection. Evidence of infection beyond the pulp at the time of fracture (defined in Table 2) and development of pulpitis also had a significant relationship. Tusk fractures were 6.7-fold more likely to develop pulpitis if either elevated white blood cell counts and/or soft tissue swelling near the orbit and/or face was present. The relationship between infection present beyond the pulp at the time of fracture and whether the tusk went on to be extracted was not significant. Descriptively, however, elephants treated with tusk extraction had approximately a 50% chance that infection was present beyond the limits of the pulp at the time of fracture. In comparison, only 15% of elephants with tusks that remained viable had evidence that infection was present beyond the pulp at the time of fracture. The majority of fractures (76%) did not have evidence that infection extended beyond the pulp at the time of fracture.

### 3.4. Fracture Treatments and Outcomes

A significant association between outcomes and treatments including endodontic intervention was observed. Tusks that received endodontic care were 12.0-fold more likely to heal than tusks treated exclusively with medical management. No difference was found between whether endodontics were utilized and whether the tusk went on to develop pulpitis, as 50% of cases developed pulpitis in both treatment categories. There was a significant association between time to initiate endodontic treatment and the development of pulpitis, with odds ratios showing fractures treated more than 48 h post-fracture were 24.2-fold more likely to develop pulpitis than fractures treated within 48 h. Time to initiate endodontic treatment had no relationship to the viability outcome of the fracture. No association existed between the use of any material as a pulp dressing and the development of pulpitis nor the outcome of the fracture. Pulp dressing materials were reported to be used the following number of times: calcium hydroxide (once), mineral trioxide aggregate (three times), formocresol (twice), and zinc oxide eugenol (twice).

Additional treatment procedures were analyzed. A significant association between the use of a tap water flush at any point in the treatment and the development of pulpitis was found, with odds ratios showing the development of pulpitis was 7.58-fold more likely when a tap water flush occurred. A similar association was not identified with the use of sterile saline (χ2 = 1.254, df = 1, P = 0.263), chlorhexidine-based rinses (χ2 = 2.476, df = 1, P = 0.116), or iodine/betadine-based rinses (χ2 = 0.248, df = 1, P = 0.619). There was no association between time to start topical treatments (within 24 h or >24 h) and the development of pulpitis nor the outcome of the fracture. Further, no relationship existed between the duration of any topical flush treatment utilized (<30 days versus >30 days) and the development of pulpitis (n = 23; χ2 = 3.569, df = 1, P = 0.059). Descriptively, however, 72.7% of those receiving topical flushes for longer than 30 days developed pulpitis, while 33.3% who received less than 30 days of flushing developed pulpitis. Numbers were not sufficient to evaluate for the impact of this variable on tusk viability. 

Regarding antibiotic choices, insufficient data precluded analysis across antibiotic classes. Very broadly, most cases that resulted in a viable tusk outcome were sensitive to the first-choice antibiotic, a broad-spectrum beta-lactam antibiotic such as amoxicillin and/or fluoroquinolone. Reports of antibacterial resistance within the data set were commonly associated with beta-lactam antibiotics such as penicillin and cephalosporin classes. In comparison, there were limited reports of resistance to fluoroquinolones. A significant association was identified between the use of antibiotics and the development of pulpitis (*n* = 25; χ2 = 9.00, df = 1, P = 0.003). Insufficient data were available to analyze the effects on time to start antibiotic therapy and the duration of antibiotic therapy on outcomes, and any relationship of outcomes to reasons to discontinue antibiotic therapy. 

The use of a non-steroidal anti-inflammatories (NSAIDs) during medical management of a Class III tusk fracture was reported in 14 out of 34 fractures. Six cases reported no development of pulpitis during short term use (8 days or less). Of the remaining eight cases, six were used only until endodontic treatment could be performed. Only two Class III fractures had ongoing management for infection that was augmented with NSAID use on an as-needed basis. Based on these reports, a relationship between NSAID use and outcomes was not pursued. 

## 4. Discussion

This study represents the first statistical evaluation of tusk fracture characteristics and management strategies in relation to outcomes of managed elephants. Based on this detailed investigation, multiple insights were gained that may help guide decision-making in the treatment of this challenging clinical condition. First, several intrinsic characteristics of the tusk fractures corresponded with outcome. As hypothesized, class of fracture was related to outcome. No Class I or Class II fractures were associated with the development of pulpitis or a tusk extraction. This finding is different from what might be expected in brachydont teeth, such as those found in humans, canids, and felids. While brachydont teeth are primarily composed of dentin, the coronal surface is covered in protective enamel and the periodontal surface is covered in cementum [24]. The structure of brachydont dentin is porous, and disruption of the overlying enamel or cementum can lead to bacterial growth within the trabeculae [25] resulting in a moderately high incidence of pulpal complications [26]. Further, though brachydont dentinal tubules are of a similar diameter to an elephant’s [24,27], the matrix of brachydont dentin is intrinsically porous even in regions sparse of dentinal tubules [24]. In contrast, the adult elephant tusk is an elodont tooth that lacks any enamel covering. The cementum layer is associated with the tusk’s periodontal attachment and can cover the entire coronal aspect [22,27], but can also erode coronally as the tusk grows beyond the gingival sulcus [28]. Though the elephant’s tusk is primarily composed of dentin (ivory), the dentin’s structure is a unique “checkered” pattern [29,30] that is a consequence of odontoblast cells moving centripetally towards the center of the pulp, depositing ivory along the way [22]. A direct comparison between the porosity of brachydont dentin and elephant dentin as it relates to pulpal pathology has not been defined to the authors’ knowledge, but the complex oscillating checkered pattern [22,30,31] that results from the 180-degree change in orientation of dentinal tubules as they form alternating helicoid patterns across microlaminal planes [27] likely promotes a more effective barrier from bacterial contamination than brachydont dentin [25]. As no Class I fractures were recorded to develop pulpitis in the data set, this hypothesis is not refuted in this study. However, it should be noted that the dentin/ivory axial and immediately rostral to the pulp chamber lacks mineralization [32]. Odontoblasts are reduced in number, concentrated towards the pulp, with truncation of the helical pattern [22,27]. This is observed as a darkened coloration towards the center of the tusk [32]. Exposure of the dentin in this region may be mistaken for pulpal involvement. It is unknown if fracture involvement of this region predisposes to pulp pathology. Further work with a larger sample size of Class I elephant tusk fractures and better definition of the proximity of those fractures to the pulp cavity would give information as to whether there is ever an indication for medical and/or endodontic intervention. No tusks in the data set were extracted due to a Class II fracture. However, there were two events in two separate Asian cows where tusks were discovered fully avulsed. Both of those females had pericoronitis evident at the time the tusks were discovered. Otherwise, the effect of disruption of the periodontal ligament (either through fracture or a disturbance in the tusk’s stability) on fracture outcomes could not be evaluated within the main data set due to the low incidence (3.8%). The one case of confirmed disruption of the periodontal ligament resolved with medical management only.

It should be noted that most of the vascular supply to the tusk is delivered via the wide apical foramen [7,15]. A second vascular supply does not enter the tooth but supplies the periodontal ligament via the surrounding alveolar bone. Intracellular fluids can subsequently flow from the periodontal ligament to the cementum and then minimally osmose into the dentinal tubules. This is the mechanism by which teeth that have undergone a root canal are able to stay functional despite losing their primary blood supply [33]. Whether there truly is a predisposition for the smaller tusks of Asian cows to be fully avulsed if periodontal disease is present is beyond the scope of this study, but information sharing among elephant managers around tusk avulsions as they occur could help build the knowledge base for this interesting phenomenon. Overall, the risks of serious complications such as pulpitis or impaired tusk viability are significantly higher in Class III fractures.

African elephants (males and females combined) were significantly more likely to develop Class III fractures than male Asian elephants, which was not expected. We also found that African elephants had more variation in the diameter of the pulp cavity exposed; 42.1% had greater than 1 cm of pulp exposed. All male Asian elephant fractures exhibited a pulp cavity exposure of less than 1 cm diameter. African elephants also had a higher propensity (64.0%) towards developing fractures within 5 cm of the gingival crest than Asian elephants (38.1%). Additionally, no Class III fractures were reported in Asian males over 10 years of age. 

Though the largest of African elephant tusks have been described to develop shortening of the pulp cavity due either to slowing of the growth rate of the tusk or to more deposition of ivory (dentin) within the pulp cavity [34], a relationship between lip circumference, age, and pulp length has not been confirmed [28]. In general, the African elephant tusk’s pulpal diameter narrows as the pulp extends rostrally [7,15]. Similar studies have not been conducted to evaluate this relationship in Asian elephants. Morphometrically, African elephants possess larger premaxillary tusk sheaths than Asian elephants [35]. Whether this corresponds to longer and/or larger pulpal canals has not been determined. The data set illustrates a propensity towards more complicated tusk fractures in African compared to Asian elephants. Whether this represents actual anatomic variation in pulp size between the species or is merely a sampling discrepancy merits further investigation as this could help elucidate whether there is a diminished risk of clinically significant tusk fractures in adult Asian elephant males. 

A significant association was demonstrated between the diameter of the pulp canal exposed and the development of pulpitis. This finding was logically expected as increased surface area exposure would create more opportunity for pulpal contamination. However, the diameter of the pulp canal exposed did not have an association with the overall outcome of whether a tusk healed or required extraction. These findings are in concordance with human dentistry literature, where a viable tooth can still be preserved even in the case of extensive pulp exposure if appropriate assessment and endodontic techniques are employed [36]. Comparatively, the presence of local infection (Table 4) at the time of fracture had a descriptive association with the onset of pulpitis, with a significant association between the presence of local infection and the outcome of tusk extraction. This finding suggests that complete removal of all diseased pulpal tissue at the time of fracture can be clinically challenging to achieve and can adversely impact outcomes. Further, a significant association existed between evidence of infection beyond the pulp at the time of fracture and the development of pulpitis, but not tusk viability. Though no mortality events were reported in the study population associated with tusk fracture complications, they have been reported in the literature [4,9]. A link between septicemia originating from the pulp can be presumed based on the robust vascular supply [7,15], though this appears to be a rarely reported cause of mortality. 

Interestingly, no association was identified between the presence of suspended pulp tissue from the fracture site and the outcome of the fracture, either in the development of pulpitis or risk of tusk extraction. In the authors’ experience, suspended pulpal tissue is very commonly associated with a disturbance in the attachment of the pulp within the pulp chamber, leading to the pulpal tissue being “suspended” from the fracture site. As discussed earlier, tusk growth is dependent on odontoblasts derived from pulpal tissue, extending towards the axis of the tusk [22]. Disruption of the pulp attachment was theorized to have a significant impact on the healing ability of the tusk. This was not observed in a statistically meaningful way, though the sample size for this variable was small. Further, this variable was asked as a “yes,” or “no” screening question, which could not account for the degree to which the pulpal tissue was both suspended and/or proximally disturbed from its attachment in the canal, or how this disturbance impacted efforts for medical management or partial pulpectomy. Additional data and more specific descriptors are necessary to assess the association between suspended pulpal tissue and tusk fracture outcomes. These findings emphasize that tusks have a remarkable capacity to heal from even serious injuries, but infection is still a risk factor to the vitality of these modified incisors. 

Other fracture characteristics were investigated, but no statistically significant associations were observed. These characteristics included the presence of dentin/ivory pearls and the age of the elephant at the time of fracture. Dentin/ivory pearls are reparative dentin that, unlike standard ivory dentin, results from mesenchymal progenitor cells that create structureless mineralized ‘stones’ more similar in structure to bone [24]. Only two cases were reported to have dentin/ivory pearls at the time of the fracture, and both went on to heal without developing pulpitis. The presence of these structures can impact effectively treating the pulp [11] by making it more difficult to effectively remove all diseased pulpal tissue. No association could be determined between age and the development of pulpitis or the ability to heal from a Class III fracture with medical management only. This was investigated briefly to assess if juvenile tusks may have an improved healing capacity compared to adults given their faster growth rate [20,34]. 

Ultimately, management strategies utilized to address Class III fractures had significant associations to the outcomes of those tusks. Most importantly, the use of endodontics in the management of Class III fractures was significantly associated with the outcome of tusk healing compared to medical management alone. Endodontic approaches to tusk repairs vary [3,6,11,33], but have a common goal of removing diseased pulp tissue and sealing off the exposed canal to further microbial contamination. In human dentistry, this procedure is defined as a partial pulpotomy [36] and the goal is to preserve the vitality of the injured tooth. Though the suffix “otomy” implies an incision made into a site, and “ectomy” alludes to excisional removal, the terms “partial pulpotomy” and “partial pulpectomy” are used similarly in both the human and veterinary literature [11,36]. Efforts to achieve a root canal in an elephant tusk are severely complicated by the depth and width of the foramen apicis dentis deep within the premaxillary sheath of the elephant’s skull [15], and the conical shape of the apex of the pulp [7,15], making full removal of the pulp impractical if not impossible. Success rates as high as 95% have been reported with partial pulpectomies in elephants [11], though severe damage to the apex of the pulp is an indication for extraction [11]. Due to the limited ability to perform diagnostics and discern the viability of the pulp at the tusk apex in elephants, apical disease was not assessed as a risk factor for extraction within the data set. Though the use of endodontics improved overall outcomes, its use did not reduce the odds of pulpitis development in comparison to medical management alone. This finding again alludes to the difficulty in achieving full removal of diseased pulp tissue due to challenges in visualization related to pulp canal depth, the presence of dentin/ivory “pearls”, or hemorrhage. Endodontics can protect the pulp from further contamination but can also entomb bacteria within the pulp if not removed before the sealing process. Though not specifically investigated, the robust vascular supply of the elephant tusk pulp suggests that the pulp should be uniquely penetrable with antibiotics. That antibiotics are commonly utilized in elephant partial pulpotomies differs from the human literature [36]. A human dental clinic is a more controlled environment than an elephant barn where these procedures occur [6], so the possibility of pulpal contamination still present after debridement and subsequent need for antibiotics is presumably reduced. Further, the ability to remove and confirm that all diseased pulpal tissue is comparatively different from elephant to human dentistry [36], as it can be complicated by the presence of reparative dentin and the limitations of radiography towards the alveolus [11]. The use of endodontics is associated with increased likelihood a tusk with a Class III fracture will remain viable. While strategies incorporating both medical management techniques and endodontic therapies were associated with improved outcomes for tusk viability compared to medical management alone, the medical management strategies (in terms of topical, antibiotic and NSAID therapy combinations) were too varied within the small subset of tusks with a “final outcome” reported to be able to make exact recommendations on which protocols definitively optimized management. However, this may be possible with continued reporting utilizing a detailed survey model. 

An unexpected finding was the significant association between the use of antibiotics and the development of pulpitis as it was surmised that antibiotics would curtail bacterial infection if present. This might be explained by a temporal association between the onset of antibiotic therapy, especially if antibiotics are predominantly started in response to evidence of pulpitis, rather than as a preventative measure if there is suspected pulp contamination. Further, it may be that the fracture cases that were not on antibiotics were less severe with no clinical indication to start this therapy. In terms of antibiotic selection choices, it was noted that Gram-negative facultative anaerobic classes of bacteria were often cultured from the pulp, with most resistance reports from beta lactam antibiotics. Pharmacokinetic studies in Asian and African elephants of oral ampicillin and fluoroquinolones demonstrate effective minimum inhibitory concentration (MIC)/half-life to undergo dosing every 12 h and every 24 h, respectively [37,38,39]. Pharmacokinetic studies are limited in the antibiotic use for dental care for elephants but have been shown effective in resolving bacterial-related foot pathology [38]. Fluoroquinolones (i.e., enrofloxacin) could be a good broad-spectrum first-choice antibiotic for suspect pulpitis, as reported resistance was limited. However, the relative paucity of information on this topic suggests that antibiotic selection in these cases should be based on culture and sensitivity results rather than empiric choices to optimize treatment outcomes. 

Studies support good to excellent pain management with NSAID use in African and Asian elephants [40]. Pharmacokinetics of commonly utilized NSAIDs within the data set, including phenylbutazone and flunixin meglumine, have been previously determined [41,42]. Use of NSAIDs can be used to reduce acute inflammation and pain upon onset of tusk fracture, but its usefulness to aid in pulpitis recovery could not be concluded based on this data set. 

Class III fractures for which endodontic treatment was not initiated within 48 h were more than 20-fold more likely to develop pulpitis, though no similar association was noted with the timing of medical therapy. However, no association was observed between timing to perform an endodontic treatment and overall outcome. This suggests that endodontic procedures performed greater than 48 h after the fracture are still beneficial to overall outcome. In association with the lack of evidence that the development of pulpitis has a negative impact on outcome of endodontic treatment pulpitis development is not a contraindication to performing a partial pulpotomy. Nonetheless, pulpitis can be a complication that can prolong management through the need for repeated endodontic revisions and/or prolonged antibiotic therapy. 

The use of tap water to rinse pulpal tissue was significantly associated with the development of pulpitis, where a similar association was not found with the use of sterile saline, chlorhexidine-based rinses, or iodine/betadine-based rinses. Though it is common practice in dental offices to utilize non-sterile distilled water in equipment, that water is unlikely a significant vehicle for bacterial introduction. Available cultures from this study showed growth of predominantly enteric bacteria from pulp cavities. The specifics of how tap water was delivered was beyond the limits of the survey, but it is likely that spigots and hoses are easily contaminated with feces in an elephant barn setting. Alternatively, the use of tap water may also have been more common in scenarios where there was a large accumulation of debris impacted within the pulp canal, necessitating a larger volume and/or higher pressure of rinsing agent than could practically be delivered via a “squirt bottle” or intravenous fluid line, as would occur with other agents. Pulpal tissue with a large degree of contamination had a higher risk of going on to develop pulpitis, as described elsewhere in the data set. Another difference in incidence could be attributed to the positive additive effect of anti-bacterial rinses in cases that did not develop pulpitis, as opposed to a negative additive effect of tap water in those that did. Finally, other environmental or behavioral variants such as pulp exposure time or repeated contamination from (ex. trunk touching/exploring) were not measured. As this study discerned only a correlation, the impact of tap water on tusk pulpal tissue and further contamination should be investigated further. Until more information is available, the authors advise judicious use of tap water on exposed pulpal tissue. 

Descriptively, a trend was observed where elephants that received topical flushing of the pulp for greater than 30 days had a greater chance of developing pulpitis compared to those treated for less than 30 days. This could allude to how the fractures that required treatment for a longer duration of time may have been more severe. These fractures and may even have had a larger pulp diameter that would take longer to seal with dentin. However, this descriptive finding also supports that prolonged topical treatment of the pulp is not particularly effective in minimizing the risk of pulpitis. The sample size to assess this variable was too small to confirm these other theorized associations. 

An unexpected finding was the lack of association between the use of a pulp dressing and the development of pulpitis. Pulp dressings are materials that are put in direct contact with the pulp that promote hard-tissue barrier formation, facilitating mechanical pulp protection [43]. Agents such as mineral trioxide aggregate and calcium hydroxide have been shown to be superior to bonding agents in achieving this goal for partial pulpotomies [43]. Further, acrylics commonly used in tusk repairs can generate heat as they polymerize, resulting in thermal necrosis to soft tissues [44], therefore giving the pulp dressing barrier a dual purpose. One case in the data set had histopathology of the pulp available postmortem that exhibited vascular congestion and acute hemorrhage. This tusk had acrylic polymer applied directly to the pulp. Therefore, while no statistically significant association was found between the development of pulpitis in the absence of a pulp dressing in our study, it is well established in small animal and human dentistry [43,45], and could be a reflection that other factors within the data set played a more significant role in outcomes. More controlled studies on the utilization of various pulp dressing materials are necessary to determine the true impact of pulp dressing on pulpal healing in elephants. 

A limitation of this survey was that it relied on records to describe tusk fracture characteristics and management. While generally thorough, such records can miss key details or vary in level of detail, which in some cases resulted in survey question answers being left blank. For example, on three occasions, survey respondents indicated that gross evidence of infection was present at the time of the tusk fracture but did not specifically state that pulpitis had developed, or adjacent infection occurred in the overall outcome of management. This leads to the question of whether there is a variance in how clinicians define pulpitis and what gross clinical signs result in diagnosis of pulpitis during management of a tusk fracture, and how those clinicians defined when pulpitis was resolved and a tusk was fully healed

The gold-standard method to confirm the presence of pulpitis is histopathological confirmation of inflammation within the pulp, as this distinguishes inflammation from other causes of pulp pathology such as thermal damage or trauma. However, thermal damage and trauma can lead to inflammation. Further, histology would be able to detect inflammation that may otherwise be too subtle to detect grossly. However, grossly evident purulent discharge or necrosis of the pulp would be sufficient to determine a diagnosis of pulpitis, visualized either on direct examination or via the aid of endoscopy [11]. Though foreign debris can be a significant nidus of contamination in the pulp, its presence would not merit the diagnosis of pulpitis per se. The aforementioned methods to diagnose pulpitis are impractical when the pulp canal is sealed. In the authors’ experience, similar to others [11], lateral, oblique and DV or VD views of the coronal aspect of tusks can aid in imaging the pulp canal and assessing for the presence of gas tracts. Lateral views of rostral portions of the alveolar bone may be possible in young elephants, but this ability quickly diminishes as the size of the skull goes beyond the limits of traditional radiography to penetrate bone. The presence of pulpal gas tracts on radiographs is proposed to be sufficient to generate a clinical diagnosis of pulpitis in a sealed pulp canal, though it would not distinguish the etiology of the pulp pathology. The presence of reparative dentin in the pulp canal would not be sufficient to establish a diagnosis of active tusk pulpitis because it represents the tusk’s response to damage to the pulpal tissue and would be present even after the injurious nidus and pulpal injury have resolved [46,47]. All of these mentioned methods to define pulpitis clinically are based on visualization and/or access to the pulp, which is not always possible when the diseased portion of the tusk is higher than the gingival sulcus and/or within the alveolar bone. As was the case with three of the Asian elephant cows in this study, pulp involvement cannot always be confirmed with the methods we currently have available. Continued use of endoscopy [11] and improved imaging technology of the alveolar bone of elephant tusks would be necessary to aid in the diagnosis of pulpitis in this clinically challenging region and should be a priority for further investigation. 

This study was unable to determine if more specific management options (such as the use of certain endodontic materials or medical treatments) were more effective than others as more variables would have subdivided the data set further. In addition, several fractures had not approached their “final” outcome at the time of survey completion, making the data set smaller. An opportunity for more consistent documentation could include creation of a formalized data sheet for managers of elephants to use when treating tusk fractures, similar to what has been developed for assessing and tracking the heart health of great apes in North American zoos [48]. In the design of this study, a goal was to develop regression equations that could determine a causal relationship between variables and their outcomes, but this could not be achieved despite the expansive efforts to generate a sufficient data set. 

The data reported here provide broad insights into tusk fracture management at a global population level. The diversity of the data limited the ability to discern specific management recommendations, but the conclusions generally align with the decision matrix advised in Management of Dental Disease in Elephants, 2019 [11]. It should be highlighted that although elephant tusks have an inherent and remarkable healing ability, overall outcomes of tusk fractures with pulp exposure were greatly enhanced with the rapid implementation of endodontic treatment. We recommend continued reporting and documentation of these veterinary procedures so that data-driven best practices can continue to be developed. These insights will aid veterinarians and dentists as they seek to develop treatment plans that minimize discomfort and optimize outcomes for managed elephants with tusk fractures.

## 5. Conclusions

Increased diameter of pulp canal exposure and the presence of infection in the pulp at the time of fracture have an association with pulpitis development, with an increased risk of tusk extraction given the latter. Tap water should be used judiciously when utilized to clean pulpal tissue and its impact on pulpal healing merits further investigation. The use of endodontic treatment versus medical management alone was significantly associated with improved outcomes (i.e., reduced risk of extraction) in tusk fractures with pulpal involvement. Continued utilization of a detailed survey model in future cases can contribute to the database necessary to understand optimal tusk fracture management strategies.

## Figures and Tables

**Table 1:** Definitions for variables pertinent to objective 2, characteristics of tusks fractured.

Variable	Definition
Presence of pericoronitis at the time of fracture	Inflammation or infection of the soft tissue surrounding the gingival attachment of the tusk.
Pulpal tissue suspended from fracture	A photograph was provided, demonstrating a fracture with pulpal tissue extending past the exposed pulpal canal.
Presence of reparative dentin	Also known as ivory pearls, dentinal bridges, and pulp stones. Elaborated that this is diagnosed via radiography and provided images of radiographs. Provided a photo where reparative dentin could be visualized in a fracture remnant.
Disruption of the periodontal ligament	Respondent was provided a diagram of the periodontal ligament for reference, and discussed that this is evidenced by tusk mobility.
Clinical evidence of infection at the time of fracture	Respondent was asked to check all that may have applied from the following list: Infection within the pulp: - Foreign debris present within the pulp. - Grossly evident purulent debris or necrosis of pulp. - Confirmation of infection via biopsy of the pulp. - Gas tracts visible within the pulp via radiography. Evidence infection extended beyond the limits of the pulp: - Elevated white blood cell counts. - Soft tissue swelling near the orbit and/or face.

**Table 2:** How fracture outcomes in the survey were classified for data analysis of objective 5 ^1^.

**No pulpitis**	**Pulpitis**
No pulpitis developed with medical management of open pulp cavity Partial pulpotomy remains intact but pulp canal has not yet sealed with dentin “Tusk never developed gross evidence of pulpitis but no radiographs confirmed absence of gas tracts; follow up fracture years later demonstrated no evidence of pulpitis” “Filling and cap are in place, we believe that no infection is present” and a fracture occurred within prior 6 months of survey response “Crown fell off [>5 years prior to survey response]. Not replaced, Tusk healed no further treatments or crown required;” and respondent reported no other evidence of pulpitis “Filling and cap are in place, we believe that no infection is present”	Pulpitis developed, and continues to be managed medically indefinitely with an open pulp cavity Pulpitis developed, and patient died from complications directly related to sepsis Pulpitis developed following partial pulpotomy; continues to be managed medically indefinitely with intact partial pulpotomy Repair or replacement of the endodontic filling following partial pulpotomy—and infection is now resolved Repair or replacement of the endodontic filling following partial pulpotomy—and infection is still present Pulpitis has developed and there are plans for tusk extraction “Tusk growth out and cut & recapped over 4 years until [radiographs] solid with no hollow, sterile (initially fistulous) tract” “Pulpitis developed, but completely cured by medical management”
**Viable**	**Non-viable, necessitating extraction**
Partial pulpotomy remained intact; canal sealed over with dentin as evidenced by radiographs Patient died due to unrelated causes; please describe the status of the healing of the tusk at the time of death by checking the box ‘other’”—if respondent elaborated that tusk fully healed based on histopathology “Pulp canal was closed by secondary dentin” “Healed with no complications/infection” “Have not done [radiograph] but assume canal has filled with dentin” and fracture occurred 6–8 months prior to survey response “Since second repair we have not done [radiograph] but assume canal has filled with dentin” and fracture occurred 7 months prior to survey response. “Tusk never developed gross evidence of pulpitis but no radiographs confirmed absence of gas tracts; follow up fracture years later demonstrated no evidence of pulpitis” “Crown fell off [>5 years prior to survey response]. Not replaced, Tusk healed no further treatments or crown required” “No pulpitis developed following medical management of open pulp cavity,” and fracture occurred 2.5 years prior to survey response, and author had opportunity to visually examine this elephant, confirming no external signs of pulpitis “Tusk never developed gross evidence of pulpitis” and fracture occurred 9 years prior to survey response “Tusk growth out and cut & recapped over 4 years until [radiographs] solid with no hollow, sterile (initially fistulous) tract. …tusk is still viable” “Tusk pulpitis developed, but was completely cured by medical management.” and fracture occurred 19 years prior to survey response	Pulpitis has developed and there are plans for tusk extraction Tusk extraction—tusk sulcus is entirely healed Tusk extraction—managing open sulcus with no focal infection/mild focal infection
	**No comment could be made on outcome**
	Tusk has a temporary cap and is awaiting endodontic repair Partial pulpotomy remains intact, but pulp canal has not yet sealed with dentin Pulpitis developed following partial pulpotomy; continues to be managed medically indefinitely with intact partial pulpotomy Patient died during anesthesia for endodontic diagnostics/procedures. Patient died due to unrelated causes; please describe the status of the healing of the tusk at the time of death by checking the box ‘other’ If response was left blank “Filling and cap are in place, we believe that no infection is present” and the fracture occurred within 5 weeks of survey response “This animal moved to another zoo…so final result is unknown”

^1^ Outcomes listed in quotations were write-in responses provided by the respondents. All outcomes not in quotations were provide as options that could be selected in the survey.

**Table 3:** The fractures of this study’s data set in relation to age, weight and species of elephant at time of fracture.

Age Class of Elephant at the Time of Fracture ^1^	*Loxodonta africana* ^2^		*Elephas maximus* ^2^		Combined sp.	
	Number of Fractures	Weight Range	Number of Fractures	Weight Range	Number of Fractures	Weight Range
Newborn juvenile (birth to 23 month)	1	560 kg (n_reported_ = 1)	0	N/A	1	560 kg (n_reported_ = 1)
Juvenile with emerging tusks (24 to 35 month)	2	843 kg (n_reported_ = 1)	3	1238 kg (n_reported_ = 1)	5	843–1238 kg (n_reported_ = 2)
Juvenile with steadily growing tusks (36 to 107 month; 3–8 year)	11	965–1502 kg (n_reported_ = 10)	8	1410–2560 kg (n_reported_ = 5)	19	965–2560 kg (n_reported_ = 15)
Sub-adults (9–17 year)	7	1952–4200 kg (n_reported_ = 6)	3	3834 kg (n_reported_ = 1)	10	1952–3834 kg (n_reported_ = 7)
Adults (18–60+ year)	9	3331–5625 kg (n_reported_ = 7)	32	2252–6140 kg (n_reported_ = 25)	41	2252–6140 kg (n_reported_ = 32)
Age or weight not reported	3	(n_weight not reported_ = 8)	0	(n_weight not reported_ = 14)	3	(n_weight not reported_ = 22)
Total	33	560–5625 kg (n_reported_ = 25)	46	1238–6140 kg (n_reported_ = 32)	79	560–6140 kg (n_reported_ = 57)

^1^ Age class based on previous definitions [14]; ^2^ combining male and female data.

**Table 4:** Analysis highlights and statistics of selected variables, organized according to this study’s objectives.

Objective	Variable Assessed	Analysis Overview	Sample Size	χ2
1. Describe individual characteristics of elephants within study	Age at time of fracture	No relationship between developmental stage of elephant and development of pulpitis in a Class III fracture No relationship between the age of elephant and whether Class III tusk fracture healed with medical management only	n_Class III fracture_ = 31 n_medical management healed class III_ = 11	χ2 = 1.777, df = 1, P = 0.183 χ2 = 1.172, df = 1, P = 0.279
2. Describe the characteristics of the tusks fractured	Class of fracture	No pulpitis or extraction reported in Class I or II fractures Class III fractures more likely to occur in African compared to Asian elephants	n_Class I or II_ = 38 n_all fractures_ = 79	χ2 = 14.820, df = 1, P ≤ 0.001.
	Shape of Class III fractures	Similar distribution between oblique & transverse	n_oblique + transverse class III_ = 27	
	Presence of pulpal tissue suspended from fracture site	Present in 33.3% of Class III fractures No association with pulpitis, tusk viability	n_Class III fractures with information on suspended pulpal tissue_ = 30	χ2 = 0.678, df = 1, P = 0.410 Pulpitis χ2 = 0.055, df = 1, P = 0.814 Viability
	Diameter of the pulp canal exposed	Larger variation in pulp canal exposure for African than Asian elephants; 100% of Asian elephants had exposure ≤1 cm Pulp canal exposures >0.5 cm more likely to develop pulpitis than canals <0.5 cm No association between pulp canal exposure and whether tusk healed or was extracted	n_Africans with reported canal diameter exposed_ = 19 n_Asians with reported canal diameter exposed_ = 5 n_>0.5 cm + <0.5 cm_ =16 n_>0.5 cm + <0.5 cm_ = 16	χ2 = 6.563, df = 1, P = 0.010 χ2 = 2.019, df = 1, P = 0.155
	Proximal extent of fracture	Descriptive difference among species, even with exclusion of Asian cows, but not significant	n_Africans_ _all and Asians males with reported proximal extent of fracture_ = 46	χ2 = 3.069, df = 1, P = 0.138
	Presence of reparative dentin within pulp cavity at time of fracture	19% of all African elephants, not reported in Asian elephants	n_Class_ _III fractures combined African and Asians_ = 32	
	Disruption of the periodontal ligament	Rarely reported	n_all_ _fractures_ = 79	
	Evidence of pulpal infection at time of fracture	Occurred frequently; 31 events Grossly evident purulent debris/necrosis of pulp was most commonly reported Fractures with evidence of pulp infection at time of fracture developed pulpitis in most cases Fractures with evidence of pulp infection at time of fracture were times more likely to result in extraction than fractures with no evidence of local infection No significant relationship between extension of infection beyond pulp at time of fracture and loss of tusk viability	n_infection events_ = 31 n_fractures with local evidence of infection_ = 20 n_fractures with viability outcome reported for this parameter_ = 19 n_fractures with viability outcome reported for this parameter_ = 20	χ2 = 4.500, df = 1, P = 0.034 χ2 = 3.767, df = 1, P = 0.052
3. Describe medical strategies utilized in fractured tusk clinical cases	Topical treatments applied to exposed pulp	Pulpitis more likely to occur if tap water was utilized during treatment	n_pulpitis outcome rep set for tap water_ = 26	χ2 = 3.909, df = 1, P = 0.048
	Period of time from the onset of fracture to the time topical treatments were started	No association between the time to start topical treatments (within 24 h or >24 h) and the development of pulpitis, or the outcome of healed versus extracted	n_onset of topical treatments data set_ = 18	χ2 = 1.778, df = 1, P = 0.182 Pulpitis χ2 = 0.093, df = 1, P = 0.761 Viability
4. Describe endodontic management strategies utilized in fractured tusk clinical cases	Protective capping procedure and/or endodontic repair utilized	Treatments including endodontic intervention were more likely to heal than tusks treated exclusively with medical management No association with use of endodontics and risk of pulpitis development	n_Class III fractures with reported final outcome_ = 22 n_Class III fractures with reported pulpitis versus no pulpitis outcome_ = 31	χ2 = 4.887, df = 1, P = 0.027 χ2 = 0.00, df = 1, P = 1
	Presence of pulp dressing	Presence of pulp dressing within endodontics had no impact on the development of pulpitis or overall outcome (healed versus extracted)	n_endodontically treated tusk with final outcome reported_ = 12	χ2 = 0.034, df = 1, P = 0.853 Pulpitis χ2 = 0.481, df = 1, P = 0.488 Viability
	Duration of time from fracture to receiving any form of endodontic treatment	Fractures where endodontic treatment was initiated more than 48 h post-fracture were more likely to develop pulpitis than sooner initiation of treatment No impact on overall outcome of extraction versus healing of tusk	n_endodontically treated tusk with final outcome reported_ = 12	χ2 = 6.000, df = 1, P = 0.014 Pulpitis
5. Outcomes	Correlate above variables to their outcomes	See above analyses		

“n” values represent the number of fractures used to analyze the variable. Variation in “n” was due to incomplete reporting for all variables and/or only including relevant data associated with the variable.

**Table 5:** Initial bacterial culture results from culture swabs and/or biopsies of exposed pulp tissue from Class III fractures.

Culture Results	Swab ^1^	Biopsy ^2^
No growth	1	1
Bacteria on cytology/biopsy and no growth	0	1
Unspecified bacterial growth	1	0
Aerobic mixed flora	1	0
Anaerobic mixed flora	1	0
Gram-positive anaerobic coccus	1	0
*Bacillus* sp.	1	0
*Corynebacterium* spp.	1	0
*Enterococcus* sp.	1	0
*Klebsiella pneumoniae*	1	0
*Proteus mirabilis*	1	1
*Proteus vulgaris*	1	0
*Pseudomonas aeruginosa*	2	0
*Serratia marcensens*	1	0
*Staphylococcus* sp.	2	0
*Streptococcus viridans*	1	0
*Streptococcus agalactiae*	1	0
*Streptococcus* sp.	1	0

^1^ Number of times this organism was cultured from an exposed pulp; collected via culture swab; ^2^ number of times this organism was cultured from an exposed pulp; collected via biopsy.

## Data Availability

Data are available for further review by contacting the primary author, J.B.R., and would involve receiving the authorization of the participating institutions (see “Acknowledgements”).

## Supplementary Materials

The following supporting information can be downloaded at: https://www.mdpi.com/article/10.3390/ani12091125/s1, Figure S1: Management and Outcomes.
