# Supplementary material for: Treatment and Outcomes of Tusk Fractures in Managed African Savanna and Asian Elephants (Loxodonta africana and Elephas maximus) across Five Continents
Source: Animals (Basel). 2022 Apr 27;12(9):1125. doi: 10.3390/ani12091125 (PMC9100196; doi:10.3390/ani12091125)
Supplement: Supplementary file 1 [file animals-12-01125-s001.zip › Supplementary File S1 Management and Outcomes.pdf]

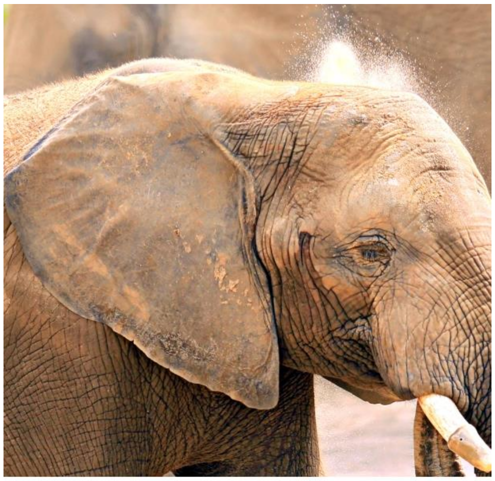

Tusk Fracture Management and Outcomes Part 1

This survey seeks to understand the different types of management that have been pursued for tusk fractures and the outcomes of those tusks since 2009. Please enter the following information for any elephant at your institution that has developed a tusk fracture that involved the pulp. This may be either exposure or a crack that communicated with the pulp cavity.

Please record the institution code you were assigned for the study

1. Species of elephant
- ◯ Asian elephant (Elephas maximus sp.)
- ◯ African elephant (Loxodonta sp.)

2. What is the stud book number for this elephant?

3. Gender
- ◯ Male
- ◯ Female

4. Birth date of elephant

DD MM YYYY

Check box if this birth date is an estimate
- ☐ Estimate

Submit

Tusk Fracture Management and Outcomes Part 2

Provide the following information *only* for the most *recent* tusk fracture for this elephant.

Page 1 of 5: Tusk Fracture Characteristics

(Please re-record this elephant's stud book number here; thank you!)

5. Date of fracture

DD MM YYYY

6. What was the approximate weight in kilograms of the elephant at the time of fracture?

7. What tusk was fractured?
- ◯ Right
- ◯ Left

8. What was the approximate length of the tusk that was fractured (prior to the fracture)?
- ◯ <1 ft (<0.3 meters)
- ◯ 1-2 ft (0.3 to 0.6 meters)
- ◯ >3 ft (>0.6 meters)
- ◯ Unknown

9. Was this tusk previously fractured?
- ◯ Yes
- ◯ No
- ◯ Unknown

10. Was any pericoronitis present at the time of tusk fracture? Pericoronitis (i.e., gingivitis) is inflammation or infection of the soft tissue surrounding the gingival attachment of the tusk.
- ◯ Yes- on the side of the affected tusk
- ◯ Yes- on the side of the UNaffected tusk
- ◯ No
- ◯ Unknown

11. Please select the class of tusk fracture that occurred. If multiple fracture types, select the highest number class that applies. (Refer to description and pictures. Diagrams modified from Weissengruber, 2005)

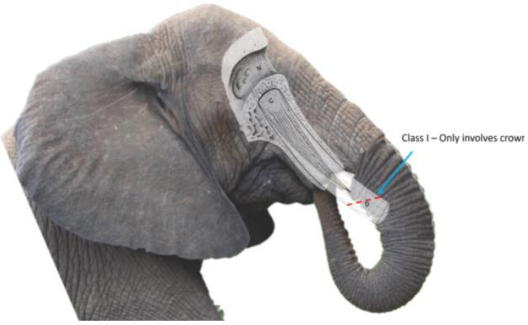

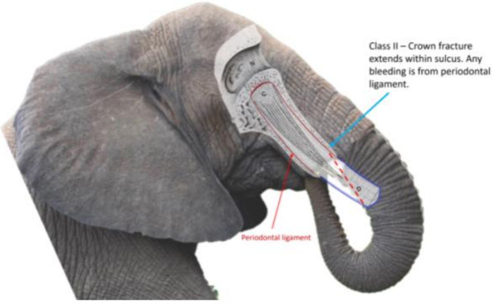

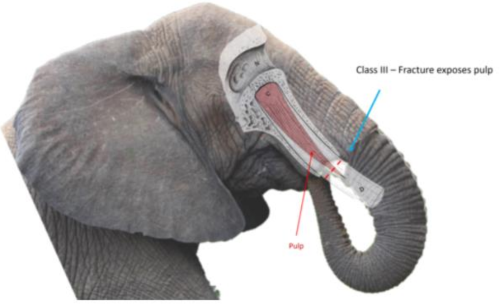

- ◯ Class I - Only involves crown
- ◯ Class II - Crown fracture extends within sulcus. Any bleeding is due to injury of periodontal ligament.
- ◯ Class III - Fracture exposes pulp
- ◯ The tusk completely avulsed out of the alveolar bone (socket). This is rare.
- ◯ Unknown

11a. If Class III fracture, what was the shape?
- ◯ Predominantly transverse to the length of the tusk
- ◯ Oblique to the length of the tusk
- ◯ Spiral fracture that did not completely break away from the tusk
- ◯ Unknown

11b. If Class III fracture, was any pulpal tissue suspended from the fracture site?

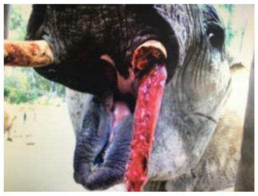

- ◯ Yes
- ◯ No
- ◯ Unknown

11c. If Class III fracture, what was the diameter of the pulp canal exposed? If oblique fracture, estimate what the diameter would have been if it were a transverse fracture.
- ◯ <0.5 cm
- ◯ 0.5 to 1 cm
- ◯ >1 cm to 2 cm
- ◯ >2 cm to 3 cm
- ◯ >3 cm
- ◯ Unknown

12. How far proximally did the fracture extend? Refer to the provided photograph for reference.

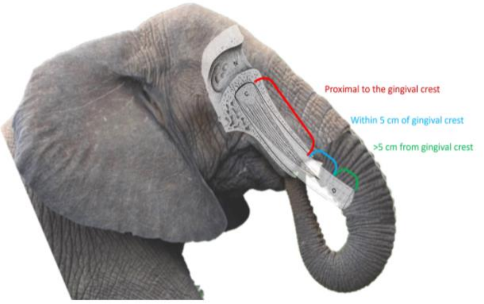

- ◯ Proximal to the gingival crest into the gingival sulcus
- ◯ Within 5 cm of the gingival crest
- ◯ Greater than 5 cm from gingival crest
- ◯ Unknown

13. Was there any reparative dentin (also known as "ivory pearls," dentin bridges, pulp stones) present within this tusk's pulp cavity at the time of fracture? This requires radiography to confirm.

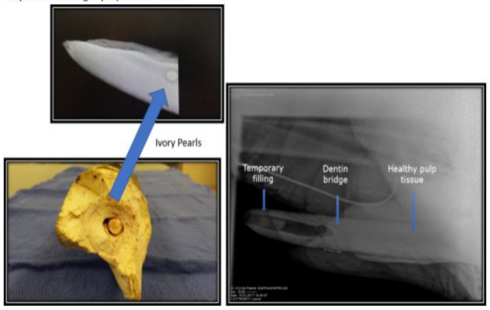

- ◯ Yes
- ◯ No
- ◯ Unknown

14. Based on physical examination, was there any disruption of the periodontal ligame (See diagram) I.e., was the tusk mobile?

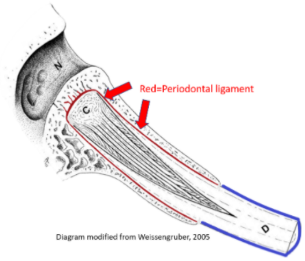

- ◯ Yes
- ◯ No
- ◯ Unknown

15. Following the fracture, was there any clinical evidence to support either local or systemic infection? Check all that apply.
- ☐ Elevated white blood cell counts
- ☐ Foreign debris present within pulp
- ☐ Grossly evident purulent debris/necrosis of pulp
- ☐ Confirmation of infection via biopsy of pulp
- ☐ Gas tracts visible within pulp via radiography
- ☐ Soft tissue swelling near the orbit and/or face

16. Please copy/paste any microbial culture and sensitivity results from a SWAB taken from the pulp and/or pulp canal. Leave blank if no cultures occurred or results are unavailable.

17. Please copy/paste any microbial culture and sensitivity results from a TISSUE SAMPLE taken from the pulp and/or pulp canal. Leave blank if no cultures occurred or results are unavailable.

Next Page

Page 2 of 5: Medical Management

18. Were any types of topical treatments applied and/or flushed over an exposed pulp/pulp cavity?

- [x] Yes
- [ ] No
- [ ] Pulp cavity was not exposed (Class I or II fracture)
- [ ] Unknown

18a. What types of topical treatments were applied and/or flushed to the pulp/pulp cavity? Check all that apply.

- [ ] Tap water
- [ ] Sterile saline/fluids flush or lavage
- [ ] Chlorhexidine-based solution and/or scrub
- [ ] Iodine and/or betadine-based solution and/or salve
- [ ] Silver sulfadiazine
- [ ] Petroleum-suspended antibiotic
- [ ] Other

18b. What was the period of time from the onset of fracture to the time topical treatments were started? Please answer in days. Write "unknown" if unknown.

18c. What was the duration that the pulp was treated topically? Please answer in days. Write "unknown" if unknown.

19. Were any antibiotics administered?

- [x] Yes
- [ ] No
- [ ] Unknown

19a. What types of antibiotics were administered?

- [ ] Sulfonamide antibiotic (ex: SMZ-TMP)
- [ ] Beta-lactam antibiotic (ex: Ampicillin)
- [ ] Nitroimidazole antibiotic (ex: Metronidazole)
- [ ] Fluoroquinolone antibiotic (ex: Enrofloxacin)
- [ ] Lincomycin antibiotic (ex: Clindamycin)
- [ ] Macrolide antibiotic (ex: Azithromycin)
- [ ] Tetracycline antibiotic (ex: Doxycycline)
- [ ] Other

19b. What was the period of time from the onset of fracture to the time antibiotics were started? Please answer in days. Write "unknown" if unknown.

19c. What was the duration that the elephant was on antibiotics? Please answer in days. Write "unknown" if unknown.

19d. If available, please record the dosages, mode of administration, and duration of any antibiotics administered; Ex: Enrofloxacin 5 mg/kg PO SID X 30 days. Write "unknown" if unknown.

19e. Were any cultured organisms resistant to your antibiotic choice?

- ( ) Yes
- ( ) No
- ( ) No culture/resistance profiles

19f. What was/were the reason(s) to discontinue treatment with antibiotics? Check all that apply.

- [ ] Sufficient period of time based on clinical judgement
- [ ] Resolution of high white-cell count
- [ ] Resolution of purulent and/or necrotic discharge from fracture site
- [ ] Resolution of radiographic evidence of pulp disease
- [ ] Resolution of inflammation/disease of sulcus
- [ ] Unknown
- [ ] Other

20a. Which of the following NSAIDs were prescribed? Check all that apply.

- [ ] Flunixin meglumine
- [ ] Piroxicam
- [ ] Ibuprofen
- [ ] Phenylbutazone
- [ ] Ketoprofen
- [ ] Unknown
- [ ] Other

20b. For what period of time were NSAIDs prescribed? Write "unknown" if unknown.

21. Were any other non-dental therapies employed for this tusk fracture?

- (•) Yes
- ( ) No
- ( ) Unknown

21a. If "other," please specify

Submit Previous

Tusk Fracture Management and Outcomes Part 3

Page 3 of 5: Endodontic Management - Temporary

(Please re-record this elephant's stud book number here; thank you!)

22. Was any type of protective capping procedure and/or restorative repair pursued on this tusk for this fracture?

- (•) Yes
- ( ) No - click "next page"

23. Was a temporary cap or covering placed to cover exposed pulp while awaiting a filling or restoration to be scheduled?

- (•) Yes
- ( ) No
- ( ) Unknown

23a. What was the time from the onset of fracture to the time that a temporary cap was placed? Write answer in days. Write "unknown" if unknown.

23b. Please elaborate on any techniques to clean/disinfect the pulp at the time of placement of the temporary cap. Check all that apply.

- [ ] Same response as "18a"
- [ ] Debridement of grossly debris-coated tissue
- [ ] Tap water
- [ ] Sterile saline/fluids flush or lavage
- [ ] Chlorhexidine-based solution and/or scrub
- [ ] Iodine and/or betadine-based solution and/or scrub
- [ ] Silver sulfadiazine
- [ ] Petroleum-suspended antibiotic
- [ ] Formalin/formaldehyde
- [ ] None
- [ ] Other

23c. Please check any hemostasis techniques for the pulp that may have been employed at the time of placement of a temporary cap. Check all that apply. Please note that any example trade names may not include all versions of this product.

- [ ] Digital pressure with cotton gauze that was then removed
- [ ] Digital pressure with cotton gauze that was left in pulp cavity
- [ ] Epinephrine solution
- [ ] Absorbable gelatin sponge (Example: Gelfoam, Vetspon)
- [ ] Oxidized regenerated cellulose (Example: Equicel)
- [ ] Buffered aluminum chloride (Example: Hemodent)
- [ ] Chitosan-based hemostatic dressing (Example: Celox)
- [ ] Ferrous/Ferric sulfate (Example: Ultradent, Vista)
- [ ] Yunnan baiyao
- [ ] Cylindrical dental cotton rolls removed from pulp cavity
- [ ] Cylindrical dental cotton rolls left in place in the pulp cavity
- [ ] Surgical laser
- [ ] None
- [ ] Unknown
- [ ] Other

23d. Please describe any material or substance that may have been used as a pulp dressing at the time the temporary cap/covering was put in place. A pulp dressing is a layer of material that is placed directly over the exposed pulp to minimize reaction of the pulp with tooth restorative/covering/capping materials (see diagram). Note that one material may be used for different purposes throughout the process of a tusk repair. Check all that apply.

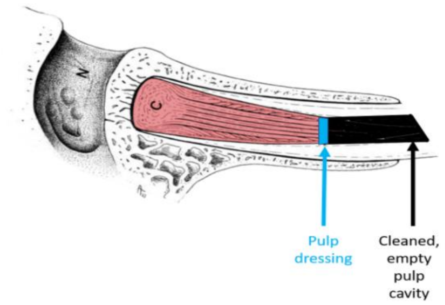

- [ ] Calcium hydroxide
- [ ] Mineral trioxide aggregate (MTA)
- [ ] Biodentine (trademark)
- [ ] Formocresol
- [ ] Ferric sulphate
- [ ] Zinc oxide eugenol
- [ ] Other
- [ ] No pulp dressing applied

23di. If more than one material or substance was used as a pulp dressing, please comment which material(s) was/were primarily in contact with the pulp.

23e. Please describe any materials that may have been used in the temporary cap/covering to cover the crack or exposed region of pulp. See diagram. Check all that apply. Note that one material may be used for different purposes throughout the process of a tusk repair.

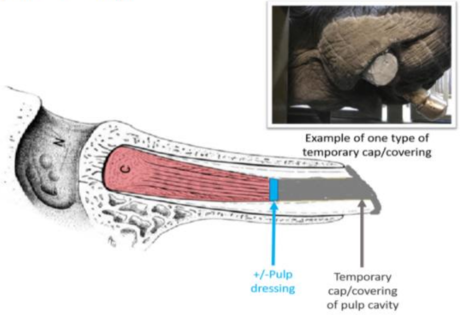

- [ ] Dental glass ionomer
- [ ] Zinc oxide eugenol
- [ ] Smooth cast 65D ie, "Marshmallow" cap
- [ ] Technovit (hoof repair)
- [ ] Plastic resin
- [ ] Epoxy
- [ ] Other

23f. How many times did the temporary cap need to be replaced? Write "unknown" if unknown.

Submit

Tusk Fracture Management and Outcomes Part 4

Page 4 of 5: Endodontic Management - Permanent

(Please re-record this elephant's stud book number here; thank you!)

24. Was a partial pulpotomy (i.e., filling or restoration) performed on the tusk?

- ◉ Yes
- ○ No
- ○ Unknown

24a. How long from the onset of fracture did a partial pulpotomy occur? Write "unknown" if unknown. Please answer in days.

24b. Please elaborate on any techniques to clean/disinfect the pulp during preparation for the partial pulpotomy procedure. Check all that apply.

- [ ] Same response as "18a"
- [ ] Debridement of grossly debris-coated tissue
- [ ] Tap water
- [ ] Sterile saline/fluids flush or lavage
- [ ] Chlorhexidine-based solution and/or scrub
- [ ] Iodine and/or betadine-based solution and/or scrub
- [ ] Silver sulfadiazine
- [ ] Petroleum-suspended antibiotic
- [ ] None
- [ ] Other

24c. Please note any hemostasis techniques utilized for the pulp at the time of the partial pulpotomy procedure. Check all that apply. Please note that any example trade names may not include all versions of this product.

- [ ] Digital pressure with cotton gauze that was then removed
- [ ] Digital pressure with cotton gauze that was left in pulp cavity
- [ ] Epinephrine solution
- [ ] Absorbable gelatin sponge (Example: Gelfoam, Vetspon)
- [ ] Oxidized regenerated cellulose (Example: Equicel)
- [ ] Buffered aluminum chloride (Example: Hemodent)
- [ ] Chitosan-based hemostatic dressing (Example: Celox)
- [ ] Ferrous/Ferric sulfate (Example: Ultradent, Vista)
- [ ] Yunnan baiyao
- [ ] Formalin/Formaldehyde/Formocresol
- [ ] Cylindrical dental cotton rolls removed from pulp cavity
- [ ] Cylindrical dental cotton rolls left in place in the pulp cavity
- [ ] Surgical laser
- [ ] None
- [ ] Unknown
- [ ] Other

24d. At the time of the debridement for the partial pulpotomy procedure, could all grossly evident necrotic and/or infected tissue be removed?

- ○ Yes
- ○ No
- ○ Unknown

24e. Did a biopsy confirm that all infected and/or necrotic tissue was removed?

- ○ Yes
- ○ No - No biopsy was collected
- ○ No - Biopsy demonstrated that there was remaining necrotic tissue sealed into the partial pulpotomy

24f. Please describe any material or substance that may have been used as a pulp dressing during the time the first partial pulpotomy for this fracture was performed. A pulp dressing is a layer of material that is placed directly over the exposed pulp to minimize reaction of the pulp with tooth restorative/covering/capping materials (see diagram). Note that one material may be used for different purposes throughout the process of a tusk repair. Check all that apply.

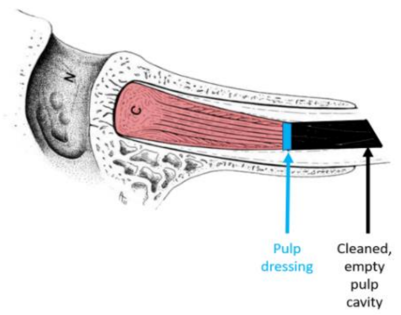

- [ ] The pulp dressing from the temporary cap/covering or a previous partial pulpotomy was still in tact and left in place
- [ ] Calcium hydroxide
- [ ] Mineral trioxide aggregate (MTA)
- [ ] Biodentine (trademark)
- [ ] Formocresol
- [ ] Ferric sulphate
- [ ] Zinc oxide eugenol (ZOE)
- [ ] Other
- [ ] No pulp dressing applied

24g. What was the composition of the permanent restoration/filling? Check all that apply. Note any bonding agents used. See diagram. Note that one material may be used for different purposes throughout the process of a tusk repair.

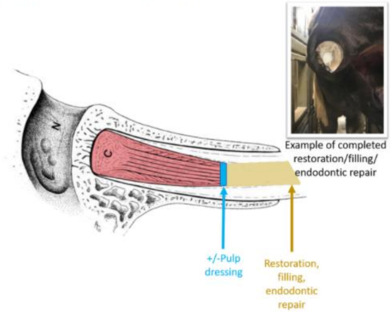

- [ ] Glass ionomer (Example: Ketac)
- [ ] Composite resin (Example: Centrix Encore)
- [ ] Technovite hoof repair
- [ ] Epoxy
- [ ] Zinc Oxide Eugenol (ZOE)
- [ ] Intermediate Restorative Material (IRM) (This is a polymer admixed with ZOE)
- [ ] Clearfil SE Bond (Trademark name: a combination bonding and primer agent)
- [ ] Gutta percha
- [ ] Unknown
- [ ] Other

24h. How many times did the partial pulpotomy restoration/filling need to be repaired/replaced following the initial placement? If none, write "0."

25. Was a permanent tusk cap/crown placed on the fractured tusk? See diagram and photo of elephant at the top of the page for reference.

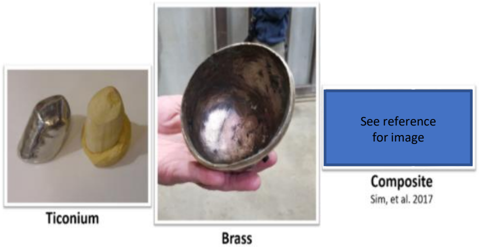

- ◉ Yes
- ○ No

25a. What material was employed for the permanent tusk cap? Check all that apply.

- [ ] Ticonium
- [ ] Brass
- [ ] Composite (like Kevlar)
- [x] Other

25ai. If "other," please specify.

25b. From the time of making the mould to the time the permanent tusk cap was placed, did the elephant go on to re-shape, chip and/or re-break the end of the tusk?

- ○ Yes
- ○ No
- ○ Unknown

25c. How was the permanent tusk cap secured to the tusk? Check all that apply.

- [ ] Epoxy
- [ ] Set screws
- [ ] Acrylic
- [ ] Glass ionomer
- [x] Other

25ci. If "other," please specify.

25d. How many times did the permanent tusk cap need to be re-secured to the tusk and/or replaced? Write "unknown" if unknown.

Next Page

Page 5 of 5: Outcomes

26. What has been the outcome thus far of the management strategy for this tusk fracture?

- [ ] No pulpitis developed with medical management of open pulp cavity
- [ ] Pulpitis developed, and continues to be managed medically indefinitely with an open pulp cavity
- [ ] Pulpitis developed, and patient died from complications directly related to sepsis
- [ ] Patient has a temporary cap and is awaiting endodontic repair
- [ ] Partial pulpotomy remained intact; canal sealed over with dentin as evidenced by radiographs
- [ ] Partial pulpotomy remains in-tact but pulp canal has not yet sealed with dentin
- [ ] Pulpitis developed following partial pulpotomy; continues to be managed medically indefinitely with in-tact partial pulpotomy
- [ ] Repair or replacement of the endodontic filling following partial pulpotomy- and infection is now resolved
- [ ] Repair or replacement of the endodontic filling following partial pulpotomy- and infection is still present
- [ ] Patient died due to complications of sepsis originating from pulpitis following endodontic repair
- [ ] Patient died during anesthesia for endodontic diagnostics/procedures
- [ ] Pulpitis has developed and there are plans for tusk extraction
- [ ] Tusk extraction - Tusk sulcus is entirely healed
- [ ] Tusk extraction - Managing open sulcus with no focal infection/mild focal infection
- [ ] Tusk extraction - Managing open sulcus with evidence of systemic infection
- [ ] Tusk extraction - Patient died due to complications from systemic infection originating from extraction site
- [ ] Tusk extraction - Patient died during anesthesia
- [ ] Patient died due to unrelated causes; please describe the status of healing of the tusk at the time of death by checking the box "other"
- [x] Other

26a. If "other," please specify

27. If the elephant died, how did postmortem findings correlate to the clinical findings? Write "animal is alive" if this question does not apply.

28. Since 2009, did this elephant develop a tusk fracture on the other tusk, and did this fracture have pulp involvement?

- ◉ Yes
- ○ No

Submit Previous
